# Family-led rehabilitation after stroke in India: the ATTEND trial, study protocol for a randomized controlled trial

**DOI:** 10.1186/s13063-015-1129-8

**Published:** 2016-01-07

**Authors:** Mohammed Alim, Richard Lindley, Cynthia Felix, Dorcas Beulah Chandramathy Gandhi, Shweta Jain Verma, Deepak Kumar Tugnawat, Anuradha Syrigapu, Craig Stuart Anderson, Ramaprabhu Krishnappa Ramamurthy, Peter Langhorne, Gudlavalleti Venkata Satyanarayana Murthy, Bindiganavale Ramaswamy Shamanna, Maree Lisa Hackett, Pallab Kumar Maulik, Lisa Anne Harvey, Stephen Jan, Hueiming Liu, Marion Walker, Anne Forster, Jeyaraj Durai Pandian

**Affiliations:** Research and Development, George Institute for Global Health India, Unit 301, Second Floor ANR Centre, Road No 1, Banjara Hills, Hyderabad, Telangana India; The George Institute for Global Health, Sydney, Australia; Sydney Medical School, University of Sydney, Sydney, Australia; Christian Medical College, Ludhiana, Punjab India; Indian Institute of Public Health, Hyderabad, India; Neurology Department, Royal Prince Alfred Hospital, Sydney, Australia; Department of Physiotherapy, Father Muller Medical College, Mangalore, India; University of Glasgow, Glasgow, UK; School of Medical Sciences, University of Hyderabad, Gachibowli, Hyderabad, India; University of Central Lancashire, Preston, Lancashire UK; The George Institute for Global Health, Oxford University, Oxford, UK; University of Nottingham, Nottingham, UK; University of Leeds, Leeds, UK

**Keywords:** Caregivers, Costs, Disability, Rehabilitation, Stroke

## Abstract

**Background:**

Globally, most strokes occur in low- and middle-income countries, such as India, with many affected people having no or limited access to rehabilitation services. Western models of stroke rehabilitation are often unaffordable in many populations but evidence from systematic reviews of stroke unit care and early supported discharge rehabilitation trials suggest that some components might form the basis of affordable interventions in low-resource settings. We describe the background, history and design of the ATTEND trial, a complex intervention centred on family-led stroke rehabilitation in India.

**Methods/design:**

The ATTEND trial aims to test the hypothesis that a family-led caregiver-delivered home-based rehabilitation intervention, designed for the Indian context, will reduce the composite poor outcome of death or dependency at 6 months after stroke, in a multicentre, individually randomized controlled trial with blinded outcome assessment, involving 1200 patients across 14 hospital sites in India.

**Discussion:**

The ATTEND trial is testing the effectiveness of a low-cost rehabilitation intervention that could be widely generalizable to other low- and middle-income countries.

**Trial registration:**

Clinical Trials Registry-India CTRI/2013/04/003557. Australian New Zealand Clinical Trials Registry ACTRN12613000078752. Universal Trial Number U1111-1138-6707.

**Electronic supplementary material:**

The online version of this article (doi:10.1186/s13063-015-1129-8) contains supplementary material, which is available to authorized users.

## Background

Stroke causes 6 million deaths each year among 17 million affected people, with the greatest burden experienced in populations of low- and middle-income countries [1]. In these countries, the burden of stroke is increasing, owing to lifestyle changes and rapid ageing of populations. Furthermore, stroke tends to affect people at relatively younger ages where there is poor control of established risk factors, in particular high blood pressure [2], with significant social and financial consequences for families, owing to limited financial protection from the costs of care and minimal social safety nets [3]. Stroke usually affects at least two people in a family, the patient and at least one family caregiver, with epidemic proportions of premature loss of productive lives in developing countries, such as India [4–6].

Like many developing countries, India is experiencing an epidemiologic transition, in which the burdens of infectious disease, maternal and child health problems are decreasing, while the burden of non-communicable chronic diseases, such as stroke and injury, is increasing [7]. In India, based upon an annual incidence of stroke of 135 to 145 per 100,000, and early case fatality of between 27 % and 41 % [7–10], it has been estimated that 1.5 million people experience stroke each year, and a further 500,000 people live with stroke-related disability. The long-term consequences of stroke on families in India, particularly in rural areas, are likely to be significant.

The most important treatment for patients with stroke is well-organized specialist care [11], which allows rapid and well-coordinated assessment and diagnosis [12, 13], early recognition and management of complications, early rehabilitation, education, and appropriate long-term support and secondary preventative therapy. Stroke unit care has greater public health impact than treatment with thrombolysis (alteplase) alone, even with the most optimal thrombolysis rates [11], because thrombolysis rates are rarely greater than 20 % (with a 5–10 % absolute benefit), yet stroke unit care is applicable to all (with a 5 % absolute benefit). Organized stroke care should be a public health priority in low- and middle-income countries, to ameliorate the increasing burden of stroke.

Although appropriate stroke unit care and rehabilitation may meet important clinical, physical and psychosocial needs during the early post-stroke phase, the needs of patients and families in the long term cannot solely be addressed in hospital [14, 15]. Advocates for early supported discharge and home-based stroke rehabilitation, which is based upon a coordinated stroke unit model of care, argue that it offers several advantages: satisfying patient choice; reducing risks (and costs) associated with inpatient care through reductions in length of hospital stay; a better rehabilitation setting, as the home setting is more focused towards realistic goals, social inclusion and a supportive environment; and leading to savings in direct and indirect costs [16, 17]. Early supported discharge provides a continuous process of rehabilitation that spans the in-hospital period and the weeks of resettlement and readjustment at home. A meta-analysis of 11 trials (mainly conducted in developed countries, where fully funded community rehabilitation teams are available) shows that early supported discharge services significantly reduced the odds of death or dependency by 21 % (odds ratio 0.79; 95 % confidence interval 0.64–0.97), without major adverse effects, either on patients or caregivers [17].

Although acute stroke units are increasing as resources improve in India, they meet the needs of only a tiny fraction of the country’s vast population, and the majority of Indians do not have access to rehabilitation services, either in hospital or following discharge. The development of effective low-cost community rehabilitation services for emerging major chronic diseases, such as stroke in India, has the potential for significant public health impact. Such interventions, if shown to be effective and affordable, could be widely scaled up or generalizable. Indeed, the research question of how to create sustainable and multiprofessional rehabilitation systems in low- and middle-income countries, including the provision of services to the rural population, was considered the second most important research priority (after equality of healthcare access) for disabled people in a recent *Lancet* expert panel [18]. Currently, most Indian stroke units are situated in the private sector [19, 20]. Clear evidence that low-cost interventions are cost-effective in India would facilitate their expansion within the public hospital system, where rehabilitation has some important features that differ from those in high-income countries: therapy is driven largely by physiotherapists, with limited input from other health professionals, such as occupational therapists; it is often poorly coordinated; and most people receive care within a large family unit (‘a joint family’, often called an extended family) after discharge [21].

A modified version of the Western model of early supported discharge, together with a development of the Indian-suggested solution based on rehabilitation delivered by a trained family caregiver, appears to be the most promising hybrid model of stroke care that could be widely implemented, if shown to be successful. Similar models have been shown to be cost-effective in the UK [22, 23]. To develop appropriate health policy, though, effectiveness and cost-effectiveness of any new model of care including rehabilitation needs rigorous evaluation in the relevant setting.

## Methods

The ATTEND study is a multicentre, prospective, individually randomized, blinded outcome assessed, controlled trial (prospective, randomized, open, blinded, endpoint design) of early supported discharge with a trained family-led caregiver.

The intervention is a stroke rehabilitation package of care that starts in hospital and continues at home, compared with usual care, in at least 1200 patients with mild to moderate disability recruited from 14 hospital sites across India.

The inclusion criteria are:

- Adults (≥18 years);
- Recent (<1 month) acute ischaemic, haemorrhagic or undifferentiated stroke;
- Residual disability (requiring help from another person for everyday activities);
- Expected to survive to discharge from hospital, with a reasonable expectation of 6 month survival (i.e. not palliative, no evidence of widespread cancer etc.);
- Able (or by proxy) to provide informed consent.

Exclusion criteria are:

- Unable to identify a suitable family-nominated caregiver for training and subsequent delivery of care;
- Unwilling or unable to adhere to follow-up.

### Randomization

**Fig. 1:**
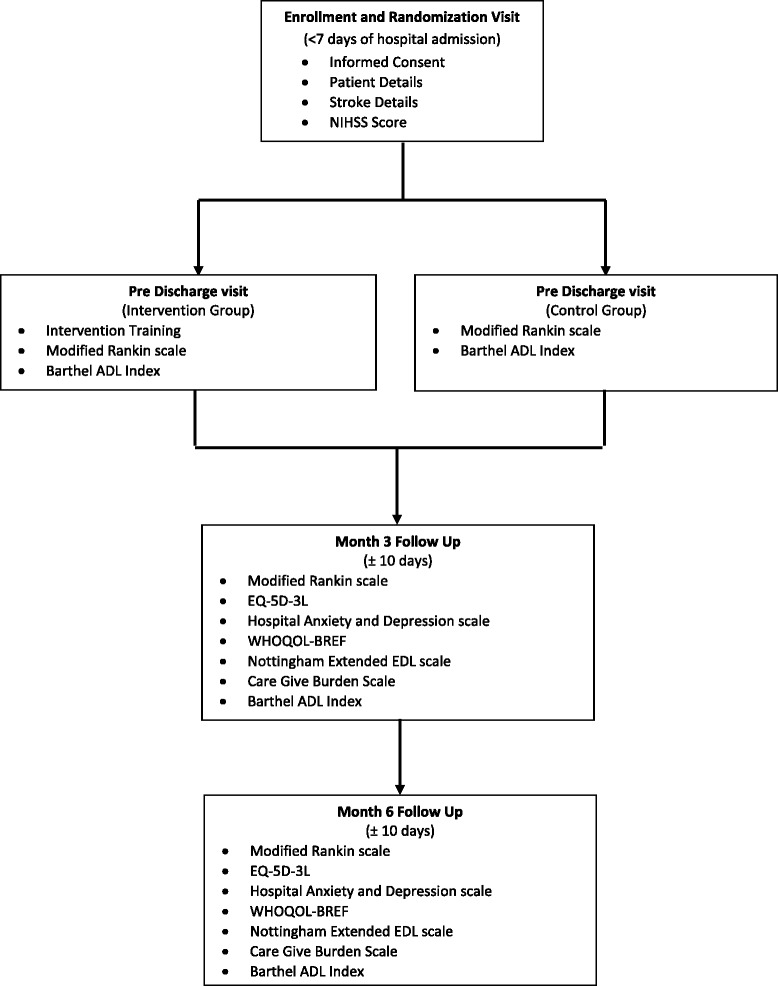
Study flow chart. ADL, activities of daily living; EQ-5D-3L, EuroQol 5-Dimensional, 3 Levels; NIHSS, National Institutes of Health Stroke Scale; WHOQOL-BREF, World Health Organization Quality of Life (Brief)

Eligible patients are identified by the trial stroke coordinator (usually a physiotherapist) and medical coordinator. A patient information sheet (Additional file 1) is shared with the patient and nominated caregiver, and outlines the study objectives and risks and benefits to the patient or caregiver. Informed consent from each participant and his or her caregiver is obtained based on the International Conference on Harmonisation Good Clinical Practices guidelines (ICH/GCP) and ethical guidelines for biomedical research on human participants published by the Indian Council of Medical Research, New Delhi (Fig. 1). If the caregiver changes by the time of the 3- or 6-month follow-up periods, the new caregiver will be asked to provide consent for the caregiver aspects of follow-up after reading the patient information sheet. Stroke patients often do not have the capacity to consent, owing to the acute effects of stroke. Capacity for informed consent is assessed by the medically qualified principal investigator at each site. The ethics committee have approved ATTEND to obtain consent from a legally acceptable representative in such cases. Once consented, patients are randomized by the trial stroke coordinator to the intervention or control arm in a 1:1 ratio within 7 days of hospital admission, using a secure, central, password-protected, web-based system, stratified by centre and stroke severity.

#### Intervention arm

Patients allocated to the intervention arm have their family-nominated caregiver trained by a specially trained trial stroke coordinator health professional (e.g., nurse, therapist) using a trial-specific structured assessment (cognition, language, function and mobility) and recommended rehabilitation package. The rehabilitation package includes a structured checklist and culturally appropriate manual (adapted to local Indian contemporaneous stroke practice) covering key activities relevant to daily living (e.g., positioning, transfers, mobilization, feeding, dressing, activity and motor practice, and monitoring of mood). Detailed instructions for selected training exercises are used from http://www.physiotherapyexercises.com. Training begins in hospital immediately after randomization for those allocated to the intervention, with a goal of approximately 60 min training per day for about 3 days, with the intention of accelerating the patient’s hospital discharge, when it is safe to do so, in addition to usual hospital care. The trial stroke coordinator visits the patient and caregiver’s home, if they are allocated to the intervention arm, on up to six occasions over the next 2 months, to provide guidance and to monitor progress after discharge, and is available by telephone for further support and guidance as the patient progresses.

A detailed written intervention guide, adapted from previous work [24], instructs all trial stroke coordinators in delivering the structured intervention in a standardized manner; this is reinforced at training sessions during the annual collaborators’ meetings.

The intervention components are:

- Information on stroke recovery trajectory, risk, identification and management of low mood, importance of repeated practice of specific activities.
- Positioning, transfers and mobility.
- Discharge planning.
- Joint goal setting with patient, nominated family caregiver and therapist (reviewed with coordinator as patient progresses and new goals set).
- Task-orientated training (particularly walking, upper limb and self-care tasks) with personalized copy of culturally appropriate manual.

The detailed intervention guide and manual are kept confidential and will only be published after the last patient follow-up has been completed, to avoid contamination of the control patients during the conduct of the trial.

#### Control arm

These patients will receive usual hospital care in terms of access to rehabilitation, timeliness of discharge and follow-up, without any explicit provision of accelerated discharge or caregiver training.

### Outcome measurement

The primary outcome measure is the effect of treatment allocation on death or dependency (a score of 0–2 on the modified Rankin scale [25]) at 6 months after randomization [26]. Patients will be seen after 3 and 6 months by an independent blinded assessor who will collect the primary and secondary outcome data.

Secondary outcome measures are:

- Effect of treatment on shift in disability, as measured by the modified Rankin scale, and analyzed with shift (ordinal) analysis;
- Answers to the simple validated recovery (Have you made a complete recovery from your stroke?) and dependency (Do you need help from another person for everyday activities?) questions [27];
- Hospital length of stay;
- Place of residence;
- Scores on the Barthel Index [26];
- Score on the Caregiver Burden Scale [28];
- Health-related quality of life (World Health Organization Quality of Life Assessment and EuroQol 5-Dimensional scores) [29, 30];
- Patient and caregiver mood (Hospital Anxiety and Depression Scale) [31];
- Extended activities of daily living (Nottingham Extended Activities of Daily Living Scale) [32];
- Health care resource use (visits to health professionals, hospitalization, and medication use) and direct costs for the patient (e.g. payment to the caregiver to act as carer for this patient, total direct costs of healthcare paid by the family since time of stroke);
- Indirect costs (e.g. family member giving up paid employment to act as caregiver) of the family;
- Direct medical costs (e.g. total expenditure during hospital admission, including first place where patient was taken, general or private admission, length of hospital stay, admission charges, investigation charges and drug treatment);
- Non-medical direct costs (e.g. travelling costs).

Clinic or telephone follow-up will be offered if home visits are not possible.

Data are collected on paper forms with an English translation on one side and the appropriate local Indian language on the other side. Each patient is identified by a unique identifier with only local sites holding the master log of names. The trial database is held and maintained by The George Institute for Global Health, with access for analysis determined by the steering committee. Future access to participant level data and statistical code will depend on additional funding to safeguard and prepare the data and is contingent upon compliance with data management guidelines in India and Australia.

#### Adverse events

Given that patients with stroke are expected to experience frequent adverse events, we defined our ‘expected’ events *a priori*. These are listed in a checklist at each follow-up. Any other adverse event is also recorded. Our expected serious events are: (1) deaths categorized as vascular (stroke, myocardial infarction, other vascular), infection, fracture, other and (2) Hospitalizations (stroke, myocardial infarction, other vascular, infection, fracture, other).

### Risks to internal validity

The main risks to the internal validity of the trial are ‘contamination’ between treatment groups, threats to the fidelity of the intervention and unblinding. To prevent ‘contamination’ between intervention and control patients in the ward during the hospital stay, we advise that the stroke coordinators delivering the intervention interview patients and carers in a private consulting or treatment room or use curtains around the patient’s bed. The time spent by the routine ward physiotherapist with control and intervention patients is monitored and recorded, to check that there is no systematic bias in routine physiotherapy. To help prevent control patients from viewing the trial manual, the manuals are given to intervention patients at the time of the first home visit and a general stroke booklet (placebo) is given to both groups. The topic of ‘contamination’ forms part of the regular training at site initiation, site visits and annual collaborators’ meetings.

Fidelity of the trial intervention is monitored during site initiation and subsequent site training visits by the clinical coordination team and a consultant physiotherapist contracted to help with training. Logs of all intervention activities are collected and analyzed to summarize the duration of each intervention and the main activities within the intervention. In addition, we will document whether the trial participants were assessed or treated by the usual routine care physiotherapists, and measure the total time spent per patient, to ensure that both intervention and control patients have the same background rehabilitation care.

Blinding is maintained by employing a dedicated blinded outcome assessor for each site. It is a requirement that the blinded outcome assessor not share the same office as the stroke coordinator and has separate computer and scanning equipment. The detailed written intervention guide has been kept confidential from the site principal investigators and blinded outcome assessors at each site. At the annual collaborators’ meetings, there are separate training sessions for the stroke coordinators and blinded outcome assessors, to maintain confidentiality of the intervention details. Patients are asked not to disclose details of home visits to the blinded outcome assessor, and intervention patients are asked to hide the trial manual when the blinded outcome assessors visit. The trial intervention is stopped one month before the first follow-up at 3 months to help reduce unblinding. Any inadvertent unblinding is recorded by the blinded assessor. Examples of unblinding are discussed at the plenary sessions at the collaborators’ meetings to share experiences, and to implement strategies to prevent future occurrences.

### Sample size and statistical consideration

In the meta-analysis of early supported discharge trials, the proportion of people dead or dependent at the end of follow-up was 50 % and the likely beneficial effect of early supported discharge treatment was an odds reduction of 21 % (95 % confidence interval 3–26 %). Therefore, the proposed minimum sample size of 1200 (600 per group) provides at least 90 % power (two-tailed α, 0.05) to detect plausible modest 10.5 % reductions in death or dependency in the intervention group with inflation by 20 % to account for patients dropping out of the trial. Ideally, a higher recruitment will allow a greater precision for treatment estimates, and could permit more detailed subgroup analysis; thus, when 1200 patients have been recruited, and if funding and time permits, the data and safety monitoring committee will advise on whether it is safe to continue recruitment. Experience during 2014 and early 2015 has allowed prediction that the trial will complete recruitment in early 2016, based on current strategies.

The intention to treat principle will be applied in all analysis. The primary endpoint measure is the proportion of those dead or dependent (modified Rankin scale score 0–2) at 6 months. This will be analyzed using an unadjusted logistic regression model. Binary secondary outcomes will be analyzed similarly, using analysis of variance (*t* tests) for continuous variables. For the shift analysis of modified Rankin scale using all seven categories (including 6 for death), ordinal logistic regression will be used, after verifying the proportional odds assumption. A statistical analysis plan will be completed prior to analysis and unblinding of the trial data.

### Ethics

Ethical approval has been obtained from Research Integrity, the Human Research Ethics Committee at the University of Sydney and at each local site (Table 1). Protocol amendments will be first approved by the University of Sydney ethics committee and then by local ethics committees before implementation. The current approved protocol is version 1.3, dated 9 December 2013.

**Table 1:** Trial sites

Collaborator	Centre	City	Name of ethics committee
Dr Jeyaraj D Pandian	Christian Medical College and Hospital	Ludhiana, Punjab	Institutional Ethics Committee
Dr MV Padma	All India Institute for Medical Sciences and Technology	New Delhi	Institute Ethics Committee
Dr PN Sylaja	Sree Chitra Tirunal Institute for Medical Sciences and Technology	Trivandrum, Kerala	Institutional Ethics Committee
Dr P Vijaya	Lalitha Super Specialty Hospital	Guntur, Andhra Pradesh	Lalitha Super Specialities Hospital Ethics Committee
Dr Sanjith Aaron	Christian Medical College	Vellore, Tamil Nadu	Office of Research Institutional Review Board
Dr Jayanta Roy	Apollo Gleneagles	Kolkata	Institutional Ethics Committee
Dr Lydia John	Baptist Christian Hospital	Tezpur, Assam	Research Ethics Committee
Dr Subhash Kaul	Nizam Institute for Medical Sciences	Hyderabad	Nizam’s Institute of Medical Sciences Institutional Ethics Committee
Dr Dheeraj Khurana	Postgraduate Institute for Medical Sciences and Research	Chandigarh	Institute Ethics Committee
Dr NC Borah	Guwahati Neurological Research Centre Hospitals	Assam	Institute of Neurological Sciences Trust Ethics Committee
Dr DS Halprashanth	Global Hospitals	Chennai	Institutional Ethics Committee
Dr B Lokesh	BGS Global Hospital	Bangalore	Institutional Ethics Committee
Dr Vivek Nambiar	Amrita Institute of Medical Sciences	Kochi	Institutional Ethics Committee
Dr Sachin Sureshbabu	St Stephen’s Hospital	New Delhi	Ethics Committee of St. Stephen’s Hospital

### Data collection and study management

Data will be collected for all patients randomized in the trial. Baseline data will be collected by the stroke coordinator and the follow-up data by the blinded outcome assessor on paper forms with appropriate local translation and are scanned and directly sent to the data management team for entry into the electronic database. The investigators and institution will allow monitors to verify the data collected on case report forms with respect to all pertinent medical records, according to ICH/GCP guidelines [33].

A data and safety monitoring committee, composed of five experts in the fields of stroke medicine, rehabilitation, statistics and clinical trials, with appropriate Indian representation, is monitoring the study, guided by a written charter with appropriate stopping rules.

The trial is governed by a steering committee formed by the applicants of the National Health and Medical Research Council (NHMRC) grant, and supplemented as agreed by the committee. The steering committee is co-chaired by Richard Lindley and GV Murthy. Other members include Jeyaraj Pandian, Pallab Maulik, Peter Langhorne, Lisa Harvey, Maree Hackett, Marion Walker, Anne Forster, BR Shamanna, Craig Anderson and Stephen Jan. The steering committee is responsible for all major decisions regarding the running of the trial, the appointment of trial staff and financial reconciliation for the NHMRC funding.

The day-to-day management of the trial is undertaken by a management committee comprised of co-principal investigators Professors Pandian and Lindley, together with Mr Mohammed Alim, the trial senior project manager (based at The George Institute, India), the trial clinical coordinator (based at the Christian Medical College Ludhiana), and representatives from the Indian Institute of Public Health, Hyderabad, together with appropriate trial administrative staff. These meetings are conducted weekly by teleconference. Trial monitoring is performed according to the monitoring plan and as per ICH/GCP, by the monitors from Indian Institute of Public Health.

The main results of the trial will be published in the name of the ‘ATTEND Collaborative Group’ with all contributions named in the primary trial manuscript. According to new NHMRC policy, this primary publication must be free to access. All publications must be approved by the steering committee with appropriate authorship determined by the steering committee and journal regulations. The NHMRC funding will be acknowledged in all publications.

## Discussion

The beneficial effects of early supported hospital discharge and home-based rehabilitation on a patient’s recovery from stroke are probably due to improved focusing of therapy around functioning and activities that are most relevant and familiar within the home environment with family support. Yet, as most of this research has been undertaken in urban settings of high-income countries, the impact in low- and middle-income countries is unclear. Moreover, uncertainty over the essential staffing and organizational requirements of such services (i.e. a complex intervention and organizational transfer) has hampered their wider implementation and development in different settings, even in the UK [34].

ATTEND is testing the effectiveness of a low-cost rehabilitation intervention that could be widely generalizable to other low- and middle-income countries. If the trial provides evidence of safety, efficacy and cost-effectiveness, it is likely that adaptations of the intervention could then be considered to augment routine care in high-income countries, with culturally appropriate adaptations to other low-income, marginalized or disadvantaged populations.

The ATTEND intervention was developed during 2010–2012 based on emerging new Indian stroke services, modified by accumulating evidence from the stroke unit and early supported discharge trials, as advised by an expert panel of stroke researchers and trial organizers. The intervention was a pragmatic culturally adapted package piloted in Ludhiana, Punjab, India [35] and was further modified based on this experience. After the main trial was funded, the clinical coordination team developed the final intervention guide with further advice from the steering committee, and a trial manual for the intervention patients was produced.

Careful thought was given to the time and cost implications for the interventions, while keeping in mind the number and quality of interventions included in the package; the stroke coordinator is trained to deliver a tailor-made package for patient-specific functional needs. The efficacy and safety of health interventions are best evaluated in randomized controlled trials and our prospective, randomized, open, blinded, endpoint study design helps ensure avoidance of bias in the follow-up of patients, a recurrent problem in previous rehabilitation trials. Extensive measures were taken to ensure that assessment was blinded in ATTEND, e.g., keeping the details of the intervention confidential to the stroke coordinators, ensuring separate training of the blinded outcome assessors, and employing dedicated research staff for the blinded assessment.

India launched its National Programme for Prevention and Control of Diabetes, Cardiovascular Diseases and Stroke in January 2008. The programme aims to strengthen infrastructure at all levels of care at the community level with the help of caregivers, who are important in the delivery of this programme, and are thus clearly aligned with a family-led rehabilitation model [36].

If ATTEND does not show efficacy or results in an unexpected hazard, data from the trial will inform the reasons why and what modifications could be made while balancing the additional costs against the infrastructure and human resource needs.

## Trial status

The first patient was randomized on 13 January 2014 and the recruitment is expected to complete by February 2016. The study recruitment is continuing as planned.

## Additional file

Additional file 1:
**Model informed consent form.** (PDF 237 kb)

## Abbreviations

GCP: Good Clinical Practice
ICH: International Conference on Harmonisation
NHMRC: National Health and Medical Research Council of Australia
