# Supplementary material for: Family-led rehabilitation after stroke in India: the ATTEND trial, study protocol for a randomized controlled trial
Source: Trials. 2016 Jan 7;17:13. doi: 10.1186/s13063-015-1129-8 (PMC4704425; doi:10.1186/s13063-015-1129-8)
Supplement: Additional file 1: — Model informed consent form. (PDF 237 kb) [file 13063_2015_1129_MOESM1_ESM.pdf]

PATIENT INFORMATION SHEET AND CONSENT FORM

Study Title

F**A**mily-led rehabili**T**a**T**ion after strok**E** in I**ND**ia **– The ATTEND trial**

Investigator Details: - **<Name>**
<Designation>
<Site Name>
<Site Address>
<Contact Number>

Introduction

You are invited to take part in this research study. The information in this document is meant to help you decide whether or not to participate in this study. Please feel free to ask if you have any queries or concerns.

If you need additional information please do not hesitate to ask. You may also wish to discuss the study with a relative or friend or your doctor. If you wish to participate in this study you will need to sign the Consent Form. By signing the Consent Form, you indicate that you understand the information and that you give your consent to participate in the study. If you do not wish to participate, you have the alternative of getting the standard treatment for your condition. Your routine follow up will be continued as per the practice.

You will be one of the 1200 patients we plan to recruit in this study across India.

Study Purpose

Stroke or brain attack is a condition which occurs when there is a blood clot which blocks the blood supply to the brain (Ischemic stroke), or it can occur as a result of bursting of brain arteries (Hemorrhagic stroke or brain hemorrhage). Stroke can affect the victim in many ways namely; sudden paralysis of one side of the body, sudden trouble speaking, sudden difficulty in walking, sudden difficulty in seeing etc.

Stroke patients face lot of difficulties in taking care of themselves. Many need help for eating, bathing, dressing, and for toileting. Person who takes care of the daily needs of the stroke patients are called care givers. Once the patients are stable the most part of stroke treatment is the rehabilitation. Patients will need Physiotherapy, Occupational therapy, speech therapy and psychological support. The best method of stroke rehabilitation in India is still unknown.

You qualify for this research because you have had a stroke. If you choose not to participate in this study or if you withdraw from the study, the care and treatment of you or your relative/husband/wife in the hospital for stroke or your relationship with the doctors or researchers will not be affected.

Study Design

This study is a multicentre, randomised, blinded outcome assessor, controlled trial in design. This means that both you as well as your investigator will know the treatment but the person who will be assessing the study outcome will not know what group you have been allocated.

Study Procedures

You will be allocated to one of the two types of treatment arms

1. Intervention arm: - While you are in the hospital your relative will be taught about various aspects of feeding, toileting, sitting, standing etc for 1-hour everyday for 3 days by the Physiotherapist. Then your relative has to carry out the treatment daily in your house.

2. Control arm: - If you are allocated to this arm you will receive standard stroke rehabilitation which includes outpatient visits to physiotherapy, occupational therapy and speech therapy departments.

Over the phone you or your relative will be called by our trial coordinator 2 times a month just to check about your progress. At 3 months and 6 months one of our staff will visit your house and document your progress. Your participation in the study will be for 6 months.

We will collect information like age, type of family, income and stroke details. All the results of the patients will be kept with strict confidentiality. You and your relative/husband/wife will not be identified in any report or publication. The information will be stored in neurology department computer which will be password protected. Only the investigators of this study will be accessing the information.

Risks or discomforts on participation

The evaluation of intervention in the study does not induce any extra risks for the patients. All patients participating in the study already had a stroke. The level of risk from study participation is unknown but it is unlikely that there are any great risks other than those associated with stroke.

Possible benefits

The results of the research may provide benefits to the society in terms of advancement of medical knowledge and/or therapeutic benefit to future patients.

Privacy, Confidentiality and Disclosure of Information

All the information collected from you for the study will be kept strictly confidential. Your information will be identified only by your unique study subject number, date of birth and initials. Only the research team investigators, institutional ethics committee and any person or agency required by law like the Drug Controller General of India to view your data, if required may have direct access to it. No report of the data or any publication of the findings will include information that would allow you to be identified.

Participation is Voluntary

Participation in this research study is voluntary. If you do not wish to take part you are not obliged to. If you decide to take part and later change your mind, you are free to withdraw from the study at any stage with no adverse consequences for you. This will in no way affect your treatment or care provided by the health care system. Before you make your decision, a member of the study team will be available to answer any questions you have.

Cost to the participant

There will be no additional cost for participation in the study. All study visits are as per the routine clinical follow up practice. You will not have any additional visits for the purpose of

the study. Thus, for participating in the study you will not receive any compensation for inconvenience and travel.

Right to new information

If the research team gets any new information during this research study that may affect your decision to continue participating in the study, or may raise some doubts, you will be told about that information.

Contact Details

If you have any problems, concerns, questions or complaints about this study, you should preferably contact

<Investigator Name>
<Designation>
<Site Name>
<Site Address>
<Contact Number>

OR

Name of the ethics committee member : ______________________
Designation : ______________________
Contact No : ______________________

OR
The Manager
Human Ethics Administration
University of Sydney
NSW 2006, Australia
Telephone +61 2 8627 8176; Facsimile +61 2 8627 8177
Email: ro.humanethics@sydney.edu.au

The study is being funded by National Health and Medical Research Council of Australia and Dr. Jeyaraj D Pandian, Professor and Head, Department of Neurology, Christian Medical College- Ludhiana is the Indian Chief Investigator for the Study.

You can also access the details of the study and other centers that are participating by visiting the website www.ctri.nic.in {Clinical Trials Registry-India (CTRI)}. This study will be registered on the same and the information would be updated at regular intervals.

Thank you for taking your time to think about participation in this study. If you wish to participate in this study you will need to sign the Consent Form provided at the end of this document.

The original signed document will remain in this hospital, a copy of this document will be provided to you.

PATIENT CONSENT FORM

Study Title
FAmily-led rehabiliTaTion after strokE in INDia – The ATTEND trial

Name of the Subject : ___________________________

Subject Initial : ___________________________

Date of Birth/ Age : ___________________________

1. I have read the Participant Information Sheet V1.3- 9 Dec 2013.
2. I have been given and I understand the information on the study concerning its nature, purpose, and duration as well as the procedures required for the project and the time involved (including any inconvenience, risk, discomfort or side effect, and of their implications) that have been explained to me, and any questions I have about the project have been answered to my satisfaction.
3. I understand that my involvement is strictly confidential and no information about me will be used in any way that reveals my identity. My medical data are strictly confidential and I authorize that they can be looked at only by persons involved in the research.
4. I understand that being in this study is completely voluntary. I am not under any obligation to consent.
5. I understand that I can withdraw from the study at any time, without any consequences immediate and the future.

Legally Acceptable Representative {LAR}

Participants who are not able to give consent due to stroke affecting the language and consciousness

Name of the LAR : ___________________________________

Relationship with the patient : ___________________________________

I consent on behalf of (Subjects Name)________________________ voluntarily to participate as a subject in this study and understand that he/she have the right to withdraw from the study at any time without in any way it affecting his/her further medical care.

-----------------------------------	------------------------------------	---------------------------------
Name of subject / LAR	Signature	Date (dd/mmm/yyyy)
----------------------------------	-----------------------------------	---------------------------------
Name of witness	Signature	Date (dd/mmm/yyyy)
----------------------------------	------------------------------------	---------------------------------
Name of Caregiver	Signature	Date (dd/mmm/yyyy)
----------------------------------	------------------------------------	---------------------------------
Name of Investigator	Signature	Date (dd/mmm/yyyy)
